# Significance of medication discontinuation on bisphosphonate-related jaw osteonecrosis in a rat model

**DOI:** 10.1038/s41598-022-25347-3

**Published:** 2022-12-12

**Authors:** Kezia Rachellea Mustakim, Mi Young Eo, Ji Hye Oh, Ju Young Lee, Hoon Myoung, Soung Min Kim

**Affiliations:** 1 Department of Oral and Maxillofacial Surgery, Dental Research Institute, School of Dentistry, Seoul National University, 101 Daehak-Ro, Jongno-Gu, Seoul, 03080 Korea; 2 Oral and Maxillofacial Microvascular Reconstruction LAB, Brong Ahafo Regional Hospital, P.O.Box 27, Sunyani, Ghana

**Keywords:** Diseases, Pathogenesis

## Abstract

Bisphosphonate (BP) discontinuation has been advised as a measure to prevent the incidence of bisphosphonate-related osteonecrosis of the jaw (BRONJ), however, its efficacy remains controversial. This study aimed to analyze the efficacy of BP discontinuation in reducing BRONJ severity following tooth extraction in a rat model. Thirty-four male Sprague–Dawley rats were divided into two BRONJ model categories: oral administration (PO) of alendronate (1 mg/kg) for 3 and 8 weeks and intraperitoneal (IP) injection of pamidronate (3 mg/kg) and dexamethasone (1 mg/kg) for 20 days. The PO model was divided into five groups (a control group without BPs and four experimental groups with 1-week discontinuation). The IP model was divided into two groups consisting of group I (without discontinuation) and group II (1-week discontinuation). One molar from both sides of the mandible was extracted. After extraction, the PO models were sacrificed at 3 and 5 weeks, and the IP models were sacrificed either immediately or at 2, 4, 6, and 8 weeks. Micro-CT showed non-significant differences among PO groups but significant differences were observed between IP groups. Most bone remodeling parameters within group I of the IP model differed significantly (*p-*value < 0.05). Histologically, group I showed a significantly higher percentage of necrotic bone than group II (51.93 ± 12.75%, *p* < 0.05) and a higher number of detached osteoclasts in TRAP staining. With discontinuation of medication for at least 1 week in rats, the effects of BPs on alveolar bone are suppressed and bone turnover and osteoclast functions are restored.

## Introduction

Bisphosphonates (BPs) are administered either orally or intravenously. Oral BPs including alendronate are most frequently prescribed for osteoporosis and osteopenia^1,2^. Intravenous (IV) BPs, such as pamidronate, are not only effective for osteoporosis, but also in treatment and management of several conditions. They are typically used for hypercalcemia, multiple myeloma, metastatic cancer, and as an alternative in patients who cannot tolerate the gastrointestinal effects of oral BPs; they also have greater bioavailability and potency^3–5^. Pamidronate, most commonly known under the brand name Aredia (Novartis Pharma AG, Basel, Switzerland), is mainly used in patients with tumors (88.24%) rather than in patients with osteoporosis (11.76%)^6^. In Korea, based on the National Health Insurance database and literature, compared to zoledronate, pamidronate is highly prescribed for osteoporosis with or without multiple myeloma and for management of multiple myeloma^7,8^.

The adverse effect of BP on jaws was first reported in 2003 by Marx^9^ and has been defined as bisphosphonate-related osteonecrosis of the jaw (BRONJ)^10^. Clinically, BRONJ is exposed necrotic bone in the oral maxillofacial region for more than 8 weeks in patients with a history of BP use with no radiation therapy^11^. In previous research and meta-analyses, the incidence of BRONJ in patients with multiple myeloma treated with pamidronate and zoledronate was reported, with the risk for developing BRONJ by pamidronate alone ranging from 0 to 18%^8,12^. According to data obtained from the FDA Adverse Event Reporting System (FAERS) from January 2004 to September 2021, cases of pamidronate were mainly reported from Africa (25.58%), followed by South America (6.65%), North America (3.58%), Oceania (2.05%), and Europe (0.51%)^6^. BRONJ pathogenesis is multifactorial, and its exact mechanism is not fully understood^5^. Our previous study using immunoprecipitation high-performance liquid chromatography to measure various effects of pamidronate and the pathophysiology of BRONJ found that pamidronate alters the expression of proteins important in wound healing and bone turnover in murine macrophages^13^. However, at present, in vivo studies to understand the effects of pamidronate in BRONJ development remains under-reported.

Tooth extraction is considered the most important risk factor in the onset of BRONJ and as a result, BP-treated patients are advised to avoid tooth extraction or to discontinue BPs before extraction^5,10,14^. The concept of discontinuing BPs before extraction is based on two ideas. First, after administration of BPs for a given time, no additional benefit is produced; second, prolonged use of BPs can give rise to adverse events^15^. To prevent BRONJ, a 2009 position paper from the American Association of Oral and Maxillofacial Surgeons (AAOMS) recommended a minimum BP discontinuation of three months before oral surgery^16^. In 2013, Damm and Jones recommended that medication should be stopped two months before oral surgery based on the BP pharmacokinetics and bone remodeling process^17^. The updated 2014 AAOMS position paper deems that the modified medication strategy of Damm and Jones^17^ is appropriate for patients at risk of BRONJ^10^.

In clinical settings, past studies have demonstrated the efficacy of discontinuing BPs by spontaneous separation and discharge of the sequestrum, symptom improvement following curettage, and higher prevalence of healed sites than in patients without BP discontinuation^18,19^.

In animal studies, mini pigs and rats have been used to assess the efficacy of IV BP discontinuation in reducing BRONJ severity^20,21^. To our knowledge, reports have not addressed the development of BRONJ in a rat model through oral and intraperitoneal BP delivery to evaluate the significance of medication discontinuation in reducing the severity of BRONJ using micro-computed tomography (micro-CT) analysis, histopathological analysis, and immunohistochemistry (IHC).

The purpose of this study was to investigate the efficacy of discontinuing BP in reducing BRONJ severity following tooth extraction in a rat model using micro-CT, histopathological, and IHC analyses. The null hypothesis of this study was that there is no significant difference in the micro-CT and histopathological findings between groups that either discontinued or continued BPs.

## Materials and methods

### Establishment of rat models for bisphosphonate-related osteonecrosis of the jaw

This study protocol was reviewed and approved by the Seoul National University (SNU) Institutional Animal Care and Use Committee (SNU-121123-12-11). All the experimental procedures were performed in accordance with the “Recommendations for Handling Regulations for Laboratory Animals for Biomedical Research” compiled by the Committee on the Safety and Ethical Handling Regulations for Laboratory Experiments of the School of Dentistry at SNU and followed the ARRIVE guidelines for animal experiments^22^.

Based on previous animal study guidelines, the power of this study is 80%^23–25^. The sample size calculation was measured using G*Power (version 3.1.9.7, Heinrich-Heine-Universitat Dusseldorf, Dusseldorf, Germany)^26^ based on the effect size of 1.57 calculated in a previous study^27^. Power analysis indicated that a minimum total sample size of 30 animals for this study has 80% power to detect an effect size of 1.57 assuming a 5% significance level and a two-sided test^26^. The principles of the 3Rs, replacement, refinement, and reduction, were applied ^28^, and a total of 34 animals was used, which was considered a suitable number to provide significant results in the study.

**Figure 1:**
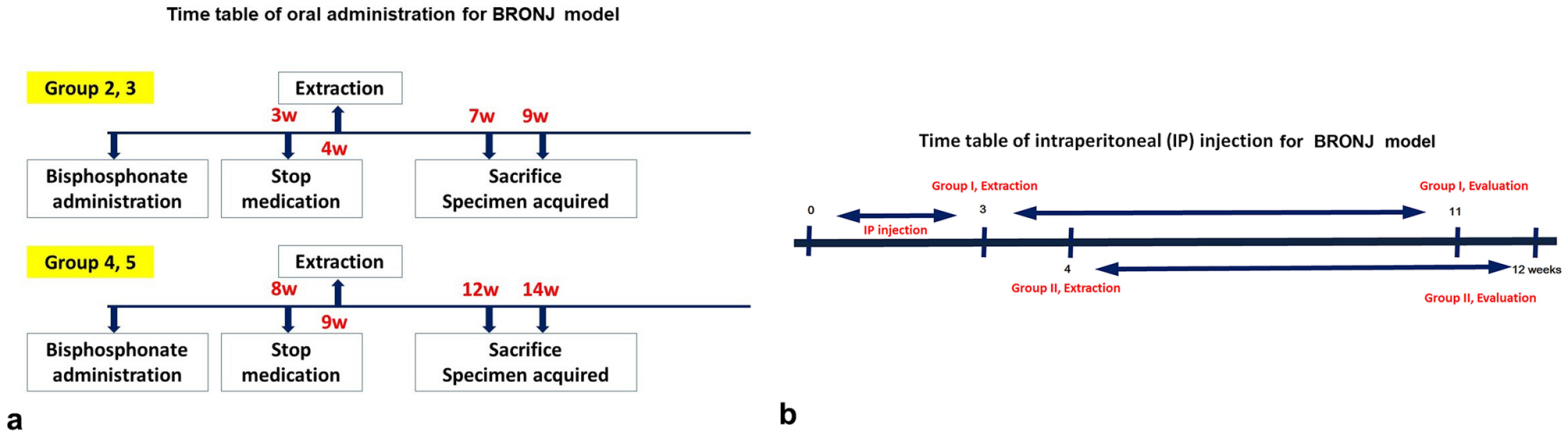
Schematic timetable for oral administration (**a**) and intraperitoneal injection (**b**) with the period of medication discontinuation in this animal study.

Thirty-four male Sprague–Dawley rats (OrientBio Inc., Seongnam, Korea) were divided into two BRONJ model categories: alendronate oral medication (PO) (10 animals, 7 weeks old) and pamidronate intraperitoneal injection (IP) (24 animals, 6 weeks old). In these experiments, the average weight of the animals for the PO model was 239.86 ± 24.74 g and for the IP model it was 141.62 ± 6.30 g. The animal experiments were conducted at the Institute for Experimental Animals, School of Dentistry, SNU. The animals were acclimatized for seven days before the start of the experiments. The animals were kept in cages, two animals were in each cage for the PO model and three animals for the IP BP model, with a 12-h light/dark cycle and ad libitum access to rodent chow and water. The experimental design is shown schematically in Fig. 1.

### Alendronate oral medication model

To establish the PO model of BRONJ, the dose of alendronate 10 mg (Fosamax^®^ tablets, MSD, Kenilworth, USA) in rats was based on 1 mg/1 kg. Furthermore, 0.22 mg of pure alendronate was dissolved in 3 ml of distilled water and orally administered via gavage to eight rats five times a week for 3 or 8 weeks, depending on the experimental group. Then, the left and right mandible molars of each animal were extracted under general anesthesia using a mixture of 90 mg/kg Ketamine^®^ (ketamine hydrochloride, Yuhan, Seoul, Korea) and 10 mg/kg Rompun^®^ (xylazine, Bayer Korea, Seoul, Korea). The extraction procedure was as follows. Each animal was positioned on the surgery table, and intraoral and extraoral disinfection was performed using betadine. The mouth was kept open by an assistant while the operator performed the extraction. The tooth was extracted gently using root forceps or curved mosquito forceps.

### Pamidronate intraperitoneal injection model

To establish the IP injection rat model of BRONJ, all the animals were given IP injections of pamidronate disodium 15 mg/mL (Panorin^®^ injection, Hanlim Pharm, Seoul, Korea) at a dosage of 3 mg/kg/day along with injections of 5 mg/mL of 0.5% dexamethasone (KGMP, Korea) at a dosage of 1 mg/kg/day for 20 days. The injections used 1 mL insulin syringes with a 23–27 G, ½ to 1-inch needle, preferably with a short bevel, and the solution volumes were 0.03 mL of Panorin^®^ and 0.17 mL of normal saline or 0.03 mL of dexamethasone and 0.17 mL of normal saline. The needle was inserted with the bevel facing up into the lower right quadrant of the abdomen toward the head at a 45° angle horizontal so that the entire bevel was within the abdominal cavity. The extraction procedures were the same as in the PO BRONJ model.

### Grouping and experimental design for discontinuation of medication

#### Alendronate oral medication discontinuation protocol

The animals were divided randomly into five groups, with two animals in each group. Group 1 served as the control group and did not receive BPs or other medication; the tooth extractions were performed 4 and 9 weeks after the initiation of the experiment. Groups 2 and 3 received 0.22 mg doses of alendronate solution for 3 weeks, and tooth extraction was performed 4 weeks after initiation of BP administration. Groups 4 and 5 received 0.22 mg doses of alendronate solution for 8 weeks, and tooth extraction was performed 9 weeks after the initiation of BP administration. The animals were sacrificed 3 and 5 weeks after the tooth extractions. BP treatment was discontinued 1 week before the extractions in all animals in the experimental groups in this model (Table 1).

**Table 1:** Animal grouping for the oral administration model. The animals were divided into a control group (group 1) and experimental groups (groups 2, 3, 4, and 5).

Group	Dose	Administration duration	Number
Group 1	None		2
Group 2	0.33 mg	3 weeks	2
Group 3	0.33 mg	3 weeks	2
Group 4	0.88 mg	8 weeks	2
Group 5	0.88 mg	8 weeks	2

### Pamidronate intraperitoneal injection discontinuation protocol

The animals were randomly divided into two groups, group I and group II. Group I contained nine animals that did not undergo discontinuation from BP before tooth extraction, with the extraction procedure performed one day after the IP injection. Group II contained 15 animals in which BP treatment was discontinued 1 week before the tooth extraction.

Two animals in group I and two in group II served as controls and were sacrificed on the same day as the tooth extraction. The other 20 animals served as the experimental subgroups and were divided evenly for 2, 4, 6, and 8 weeks of observation between extraction and sacrifice (Table 2).

**Table 2:** Animal grouping for the intraperitoneal injection model. The animals were divided into group I and group II.

Group		Injection (IP)	Duration	Extraction	Sacrifice	Number
Group I	Control	Panorin 3 mg/kg/day + Dexa 1 mg/kg/day	Daily for 20 days	On the last day of injection	0 weeks	n = 2
	Experimental	Panorin 3 mg/kg/day + Dexa 1 mg/kg/day	Daily for 20 days	On the last day of injection	2, 4, 6, 8 weeks	n = 7
Group II	Control	Panorin 3 mg/kg/day + Dexa 1 mg/kg/day	Daily for 20 days	1 week after last injection	0 weeks	n = 2
	Experimental	Panorin 3 mg/kg/day + Dexa 1 mg/kg/day	Daily for 20 days	1 week after last injection	2, 4, 6, 8 weeks	n = 13

The BP administration and discontinuation periods for the rats were established through correlations between rat and human age in which one human year is equivalent to 13.8 days (2 weeks) in rats^29^. Therefore, 20 days of injections in rats represent one and a half years in humans, and a 1-week discontinuation of medication in rats represents 6 months of medication discontinuation in humans.

Blood samples were collected from the tail vein 1 week after the first injection; the last day of injections; the day of extraction; and 2, 4, 6, and 8 weeks post-extraction. All animals were euthanized by CO_2_ inhalation. The mandibles of the rats were harvested and fixed in 10% formalin for micro-CT and histopathological analyses.

### Micro-CT analysis of medication discontinuation

The obtained mandible specimens were analyzed using high-resolution micro-CT scanning on a Skyscan 1172^®^ (Bruker, Kontich, Belgium). For the PO model, the imaging parameters were adjusted to an 80 kV source voltage, 124 μA source current, and 14.87 μm image pixel size, with a 0.5 mm aluminum filter and 0.4° angular steps. For the IP model, the imaging parameters were adjusted to a 70 kV source voltage, 141 μA source current, and 14.87 μm image pixel size, with a 0.5 mm aluminum filter undergoing a 360° rotation with 0.3° steps.

Following the scanning procedure, the datasets were reconstructed using NRecon 1.6.9.8^®^ (Bruker, Kontich, Belgium) software. The reconstruction settings were adjusted with smoothing set to 3, ring artifact reduction set to 6, and beam hardening correction set to 20%.

**Figure 2:**
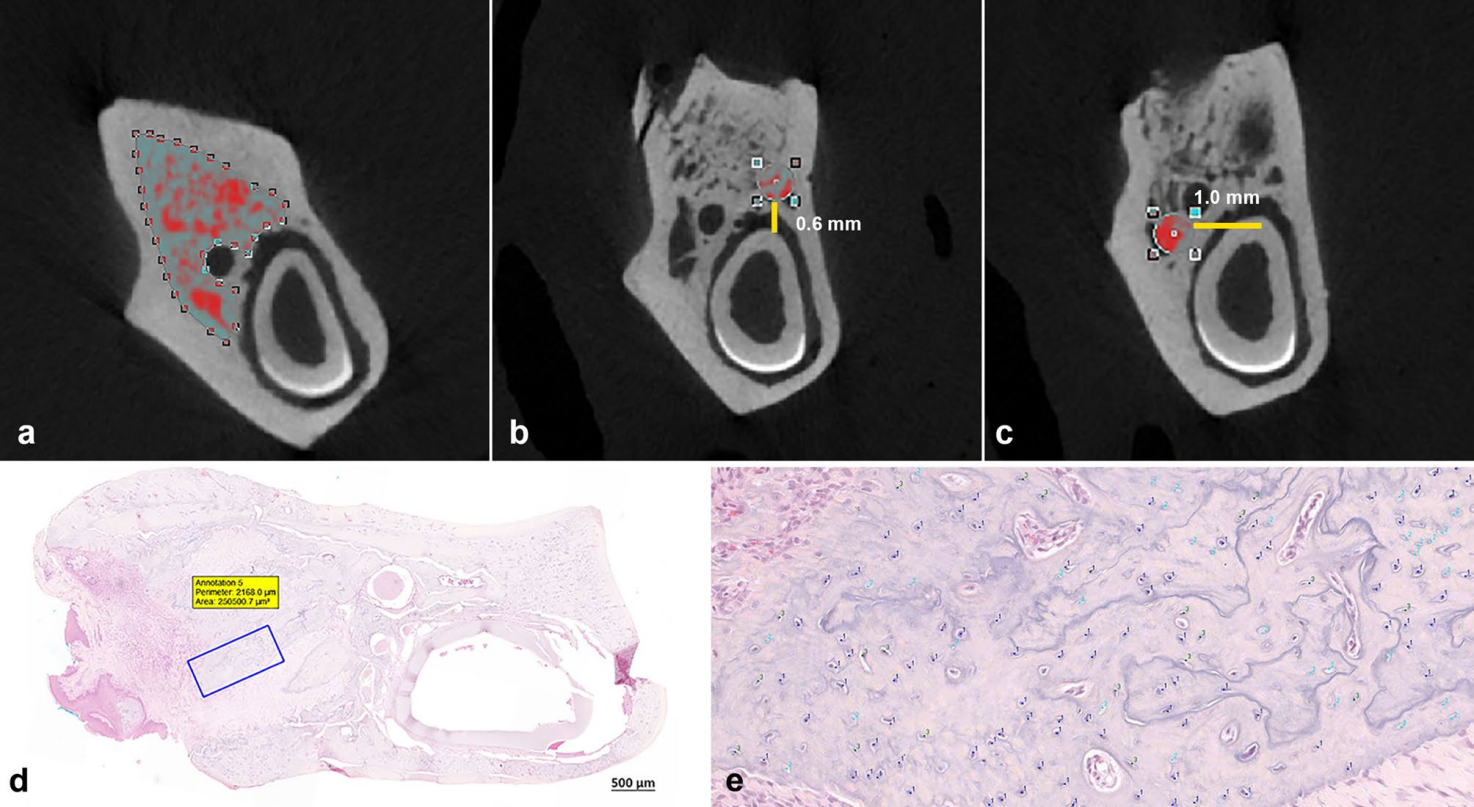
Micro-CT analysis. Manual delineation of the region of interest (ROI) encompassing the trabecular bone surrounding the extraction socket (**a**). Standardized round ROI (0.52 × 0.52 mm^2^) containing trabecular bone in the lingual portion of the mandible, with 0.6 mm of vertical distance from the incisor (**b**). Standardized round ROI at the buccal portion of the mandible, with 1.0 mm of horizontal distance from the incisor (**c**). Cell quantification for measuring the necrotic bone indicator percentage. An area of interest was established using a fixed rectangular form of 0.25 mm^2^ within the bone surrounding the extraction socket in all specimens at a magnification of ×20 (**d**). Fiji ImageJ (version 1.53c; NIH, Maryland, USA) was used to quantify the cells using cell counter plugins type 1 (osteocytes), type 2 (empty lacunae), and type 3 (pyknotic osteocytes) (**e**).

Each dataset was opened and adjusted using DataViewer^®^ (DataViewer, Brukerm Kontich, Belgium) software and the image analysis was performed using CT-Analyser (CTAn) Software^®^ (version 1.7.0, Bruker, Kontich, Belgium). The regions of interest (ROIs) were determined in the coronal plane and defined in three areas: (1) manual delineation encompassing trabecular bone surrounding the extraction socket, (2) standardized 0.52 × 0.52 mm^2^ round ROI containing the trabecular bone close to the lingual surface of the mandible (1.0 mm from the incisor), and (3) standardized 0.52 × 0.52 mm^2^ round ROI containing trabecular bone close to the buccal surface of the mandible (0.6 mm from the incisor) (Fig. 2a–c). The images were segmented into binary images using a Gaussian filter and a fixed threshold with a lower value of 70 and an upper value of 224 to extract the mineralized tissues. The effects of BPs on bone mineral density (BMD, g/cm^3^), bone volume fraction or bone volume/tissue volume (BV/TV, %), bone-specific surface or bone surface/volume ratio (BS/BV, 1/mm), bone surface density or bone surface/tissue volume (BS/TV, 1/mm), trabecular thickness (Tb.Th., mm), trabecular number (Tb.N., 1/mm), and trabecular separation (Tb.Sp., mm) were measured and compared between groups (Fig. 5). The three-dimensional images of the dataset were reconstructed using CTVol software^®^ (SkyscanVR, Kontich, Belgium).

### Histological and immunohistochemical analyses of medication discontinuation

The samples from each group were trimmed and decalcified using 0.5 M ethylene diamine tetra-acetic acid (EDTA, Biosesang, Seongnam, Korea) at pH 8.0 for 10 days, dehydrated using 70% ethanol, fixed in 10% formalin-buffered solution, and embedded in paraffin wax. The 4 μm thick slides were prepared and cleaned with xylene for 10 min, followed by staining with hematoxylin and eosin (H&E) and Masson’s trichrome (MT). The histological slides were scanned with a 3D Scan Panoramic Histech scanner^®^ (3D Histech. Budapest, Hungary) and examined using CaseViewer^®^ (version: 2.0, 3DHISTECH, Budapest, Hungary). For quantitative analysis, the necrotic bone indicator (NBI) percentage was quantified as the ratio of empty osteocyte lacunae and pyknotic osteocytes to the total number of lacunae in the field. An area of interest was established in all specimens using a fixed rectangular form of 0.25 mm^2^ within the bone surrounding the extraction socket at a magnification of 20×. Fiji ImageJ^®^ (version 1.53c; NIH, MD, USA) was used to quantify the cells using cell counter plugins type 1 (osteocytes), type 2 (empty lacunae), and type 3 (pyknotic osteocytes) (Fig. 2d,e).

For immunostaining, paraffin-embedded tissues were cut at 4 μm intervals and transferred onto microscope glass slides using a semi-automated rotary microtome (Leica Biosystems, IL, USA). Every microscope slide was evaluated under a BX51^®^ light microscope (Olympus Co., Tokyo, Japan) to confirm all the tissue structures present. Vascular endothelial growth factor A (VEGFA) and cluster of differentiation 31 (CD31) were used to evaluate angiogenesis. Tumor necrosis factor alpha (TNF-α) and interleukin 6 (IL-6) were used as inflammatory markers, and tartrate-resistant acid phosphatase (TRAP) and alkaline phosphatase (ALP) were used as bone turnover markers. Staining was scored semi-quantitatively with the immunoreactive score (IRS) system described previously^30^. The IRS gives a range of 0–12 as a product of multiplying the positive cell proportion score (0–4) and the staining intensity score (0–3), in which 0–1 is negative, 2–3 is mild, 4–8 is moderate, and 9–12 is strongly positive cell staining. For TRAP, the numbers of attached and detached osteoclasts on the whole slide were counted.

### Statistical analyses of micro-CT and histological data of medication discontinuation

All statistical analyses were performed using IBM SPSS^®^ software (version 26.0, IBM Corp, Armonk: NY, USA). The means and standard deviations were obtained for BMD, bone morphometry parameters, and quantified cells. The obtained data were tested for normality using the Shapiro–Wilk test. One-way analysis of variance was used for comparisons between subgroups, within one group, and among multiple groups. The independent samples t-test was used for comparisons between two groups and *p* values < 0.05 were considered statistically significant.

### Ethics approval

This study protocol was reviewed and approved by the Seoul National University (SNU) Institutional Animal Care and Use Committee (SNU-121123-12-11). The experiment was in accordance with the “Recommendations for handling of Laboratory Animals for Biomedical Research” and complied with the Committee on Safety and ethical Handling Regulations for Laboratory Experiments at SNU. Animal studies were conducted following the ARRIVE guidelines and are in accordance with the 1964 Helsinki declaration and its later amendments or comparable ethical standards.

## Results

### Establishment of oral and intraperitoneal BP-medicated rat models for bisphosphonate-related osteonecrosis of the jaw

**Figure 3:**
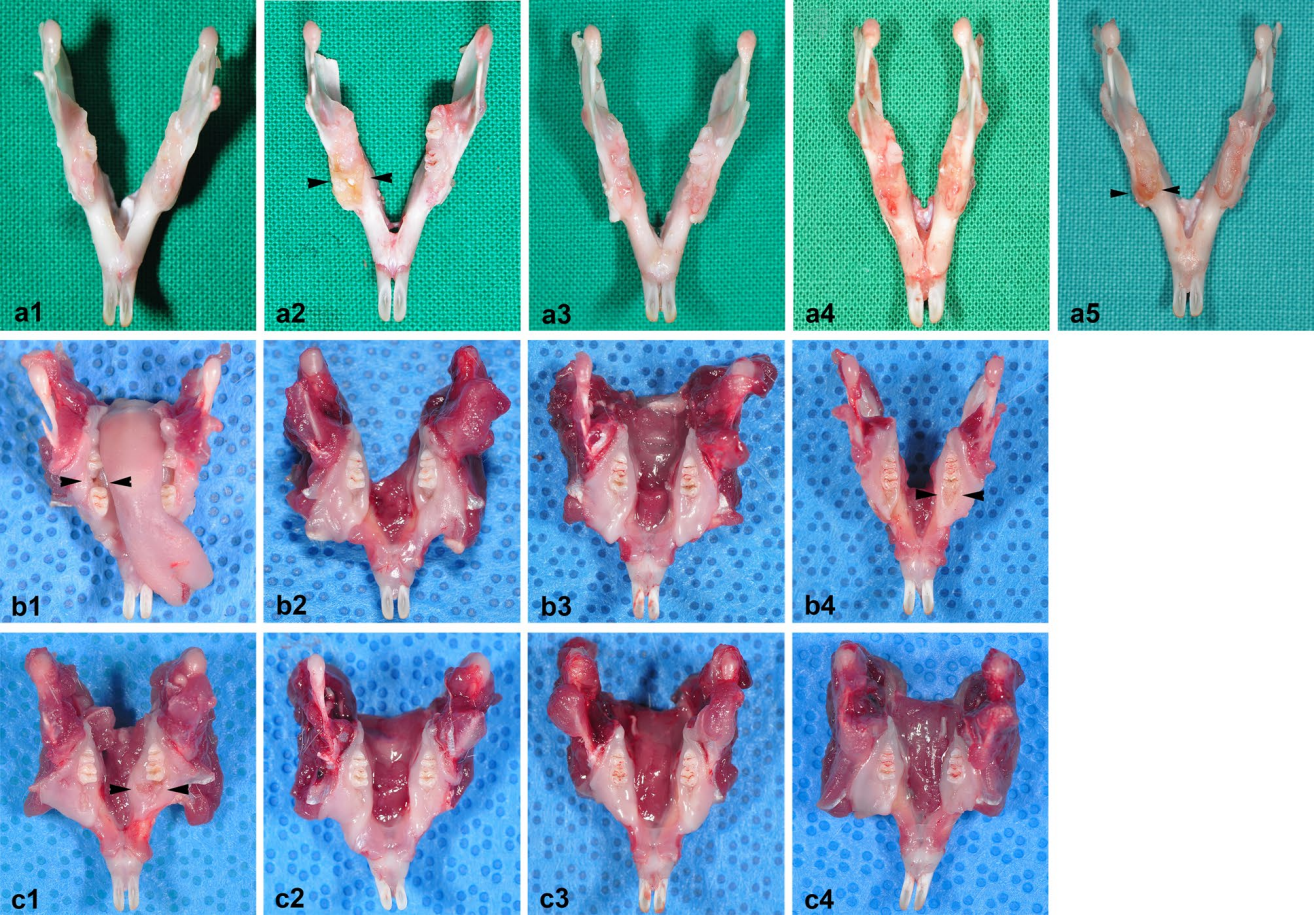
Clinical views of specimens obtained from the PO BRONJ model. Uneventful healing was observed in group 1 (**a1**). In the clinical view of group 2, inflamed tissue can be seen in the extraction socket area (arrowheads) (**a2**). In the clinical view of group 3, the extraction socket was fully healed, with irregular tissue formations compared with the control group (**a3**). In group 4, wound healing on the extraction socket is visible, but the retained root can also be seen (**a4**). In group 5, inflamed tissue is visible on the extraction socket (arrowheads) (**a5**). Clinical views of specimens obtained from the IP BRONJ model. The specimens obtained from group I (**b1**–**b4**). Two weeks post-extraction, exposed necrotic bone was observed (arrowheads) (**b1**). Representative clinical views at 4 weeks and 6 weeks post-extraction (**b2**,**b3**). Incomplete wound healing with dehiscence and bony exposure was observed 8 weeks post-extraction (arrowheads) (**b4**). The specimens obtained from group II (**c1**–**c4**). The representative clinical view taken 2 weeks post-extraction reveals mucosal ulceration surrounding the extraction area (arrowheads) (**c1**). Uneventful wound healing was observed 4, 6, and 8 weeks post-extraction (**c2**–**c4**).

We observed wound healing in and around the extraction sockets in the PO and IP BRONJ models (Fig. 3). In the PO BRONJ model, the control group showed uneventful healing after extraction. Inflamed tissues were observed in groups 2 and 5. In all specimens of the PO model, no exposed necrotic bone was observed.

In the IP BRONJ model of the subgroups sacrificed at 2 weeks, exposed necrotic bone was observed in group I whereas only mucosal ulceration was observed in group II. In the 8-week subgroups, dehiscence and bony exposure were observed in group I, but not in group II. Pamidronate disodium 15 mg/mL at a dosage of 3 mg/kg/day along with 5 mg/mL 0.5% dexamethasone injection at a dosage of 1 mg/kg/day for 20 days without BP discontinuation was effective in creating a repeatable BRONJ model in rats. Two animals died due to improper handling during the experiment and were excluded from the study. One animal died from severe abdominal distension due to trauma from the IP injection. Necropsy results revealed ascites around the intestines indicating leakage of the medication agents from a ruptured cecum. Based on the blood test, we also found a high percentage of monocytes: 36.6% (reference range 0–4.1%) indicating infection^31^. The literature mentions that a poorly administered IP injection in a rodent can result in lesions of the internal organ, peritonitis, and death due to bacteremia and septic shock^32^. Another animal died of an unsecured airway during retraction, resulting in a blocked upper airway.

### Clinical evaluation of medication discontinuation with blood tests

**Figure 4:**
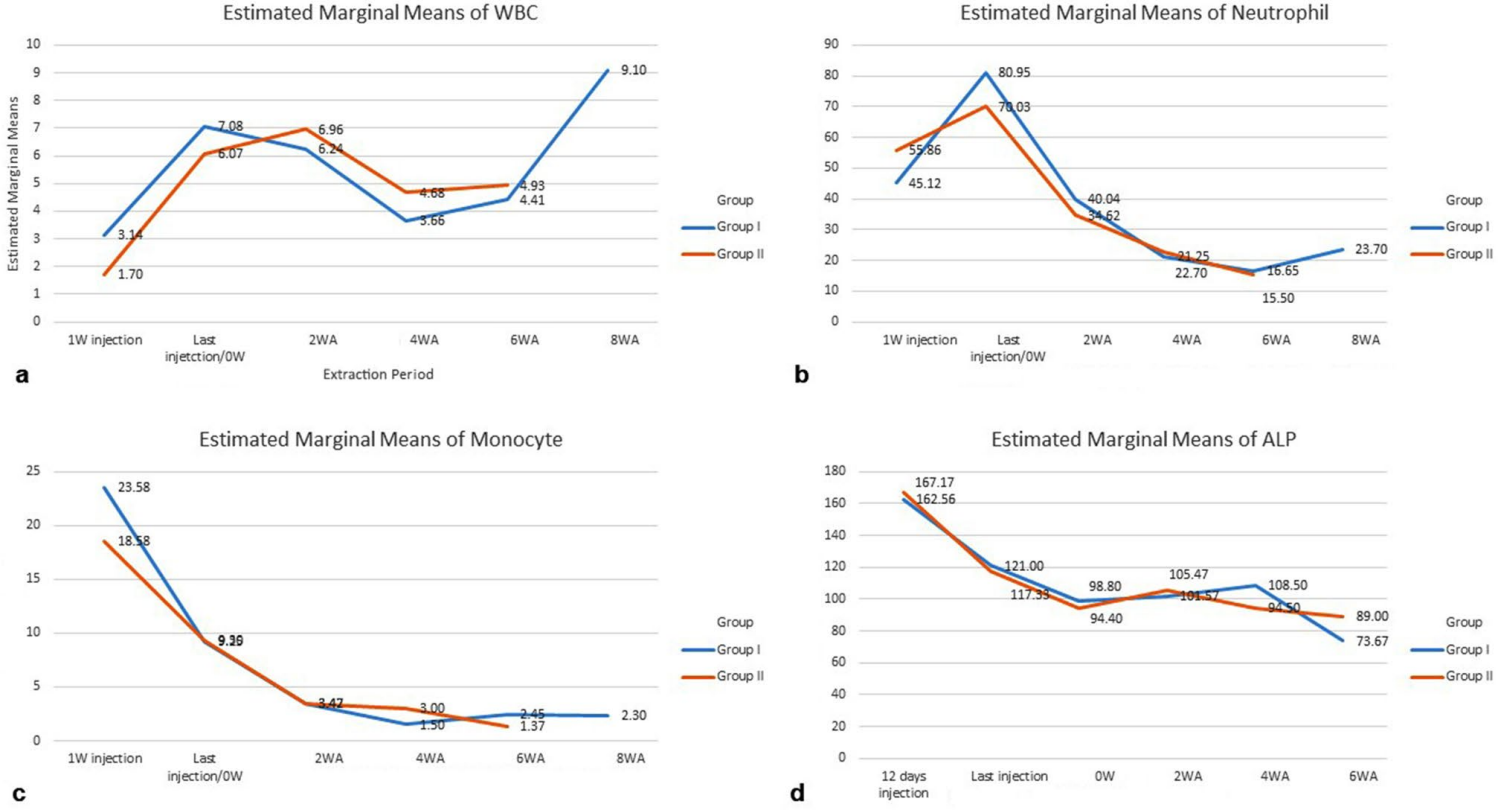
Laboratory results of blood testing for white blood cells (**a**), neutrophils (**b**), monocytes (**c**), and ALP (**d**) show changes in the status of inflammatory markers after the administration of pamidronate in the developing BRONJ model.

The level of white blood cells (WBCs) increased from 1 week after injection to the last injection in both groups I and II. Two weeks post-extraction, the WBC level decreased and reached a plateau by 4 weeks post-extraction in group II. Meanwhile, in group I, the WBC level decreased 2 weeks post-extraction and then increased dramatically by 4 weeks post-extraction (Fig. 4a). The neutrophil level also increased from 1 week of the injection to the last injection in both groups and then tended to decrease from the last injection to the end of the experiment (Fig. 4b). The monocyte level tended to decrease over time in both groups (Fig. 4c). The ALP level decreased following the injections in both groups, but in group I, it increased 2 weeks and 4 weeks post-extraction and then decreased dramatically. Meanwhile, in group II, the ALP level increased at 2 weeks post-extraction, decreased at 4 weeks post-extraction, and mostly plateaued at 6 weeks post-extraction (Fig. 4d).

### Micro-CT results of medication discontinuation

#### Medication discontinuation in the alendronate oral model

The BMD (g/cm^3^) and bone morphometry parameters (BV/TV, %; BS/BV, 1/mm; BS/TV, 1/mm; Tb.Th., mm; Tb.N., 1/mm; and Tb.Sp., mm) were compared between the control group and the experimental groups. Among the five groups, group 3 showed the highest BMD (0.887 ± 0.108 g/cm^3^). Based on the duration of alendronate administration, the highest BMD was found in the control group (0.858 ± 0.039 g/cm^3^), followed by the group that received 3 weeks of alendronate administration with a BP discontinuation of 1 week. However, the results do not differ significantly (Table 3).

**Table 3:** Bone mineral density (BMD) in oral administration model groups showing the effect of bisphosphonate on the bone in groups with a medication discontinuation compared with the control group.

BMD (g/cm^3^)			
Group	Mean ± SD	Drug administration duration	Mean ± SD
Group 1	0.858 ± 0.039	Control	0.858 ± 0.039
Group 2	0.792 ± 0.057	3 weeks	0.840 ± 0.089
Group 3	0.887 ± 0.108		
Group 4	0.839 ± 0.027	8 weeks	0.824 ± 0.032
Group 5	0.808 ± 0.032		
*p*-value	0.251		0.568

The statistical analysis was performed using ANOVA. The data are depicted as the mean ± standard deviation. *BMD* bone mineral density.

In terms of bone morphometry, group 3 had the highest BV/TV (68.989 ± 13.188%), BS/BV (16.392 ± 1.734/mm), and Tb.N. (4.697 ± 0.693/mm), whereas group 2 had the highest BS/BV (33.378 ± 16.064/mm). Tb.Th. was highest in group 4 (0.153 ± 0.013 mm), and group 5 had the highest Tb.Sp. (0.180 ± 0.243 mm) (Table 4). Based on the duration of drug administration, the control group had the highest BV/TV (63.725 ± 5.032%) and Tb.Th. (0.149 ± 0.119 mm). Three weeks of alendronate administration with medication discontinuation for 1 week produced the highest BS/BV (29.253 ± 10.638/mm) and Tb.Sp. (0.171 ± 0.010 mm), and Tb.Sp. was lowest after 8 weeks of alendronate administration with 1-week BP discontinuation (0.122 ± 0.550 mm). Furthermore, BS/TV and Tb.N. were highest in the group that received 8 weeks of treatment (16.392 ± 1.734/mm, 4.697 ± 0.693/mm, respectively). However, those results do not differ significantly (Table 4).

**Table 4:** Micro-CT bone morphometric parameters from the oral administration model groups showing the effects of bisphosphonate on the bone in the groups with a medication discontinuation compared with the control group.

Group	BV/TV (%)	BS/BV (1/mm)	BS/TV (1/mm)	Tb.Th. (mm)	Tb.N. (1/mm)	Tb.Sp. (mm)
Group 1	63.725 ± 5.032	25.150 ± 2.351	15.568 ± 1.075	0.149 ± 0.119	4.289 ± 0.350	0.151 ± 0.039
Group 2	51.698 ± 15.156	33.378 ± 16.064	15.702 ± 3.518	0.138 ± 0.411	3.779 ± 1.035	0.171 ± 0.010
Group 3	68.989 ± 13.188	25.128 ± 3.658	16.392 ± 1.734	0.145 ± 0.009	4.697 ± 0.693	0.122 ± 0.550
Group 4	62.506 ± 3.348	24.032 ± 2.366	14.886 ± 0.932	0.153 ± 0.013	4.111 ± 0.184	0.160 ± 0.0157
Group 5	58.599 ± 3.988	24.539 ± 0.647	14.217 ± 0.826	0.152 ± 0.005	3.868 ± 0.313	0.180 ± 0.243
*p-*value	0.205	0.356	0.471	0.855	0.081	0.319
Control	63.725 ± 5.032	25.150 ± 2.351	15.568 ± 1.075	0.149 ± 0.119	4.289 ± 0.350	0.151 ± 0.039
3 weeks	60.343 ± 15.304	29.253 ± 10.638	15.702 ± 3.518	0.138 ± 0.411	3.779 ± 1.035	0.171 ± 0.010
8 weeks	60.553 ± 3.997	24.286 ± 1.628	16.392 ± 1.734	0.145 ± 0.009	4.697 ± 0.693	0.122 ± 0.550
*p-*value	0.797	0.340	0.471	0.855	0.081	0.319

The statistical analysis was performed using ANOVA. The data are depicted as the mean ± standard deviation. *BV/TV* bone volume/total volume (bone volume fraction), *BS/BV* bone surface/segmented bone volume (specific bone surface), *BS/TV* bone surface/total volume (bone surface density), *Tb.Th.* trabecular thickness, *Tb.N.* trabecular number, *Tb.Sp.* trabecular separation.

#### Medication discontinuation in the pamidronate intraperitoneal injection model

**Figure 5:**
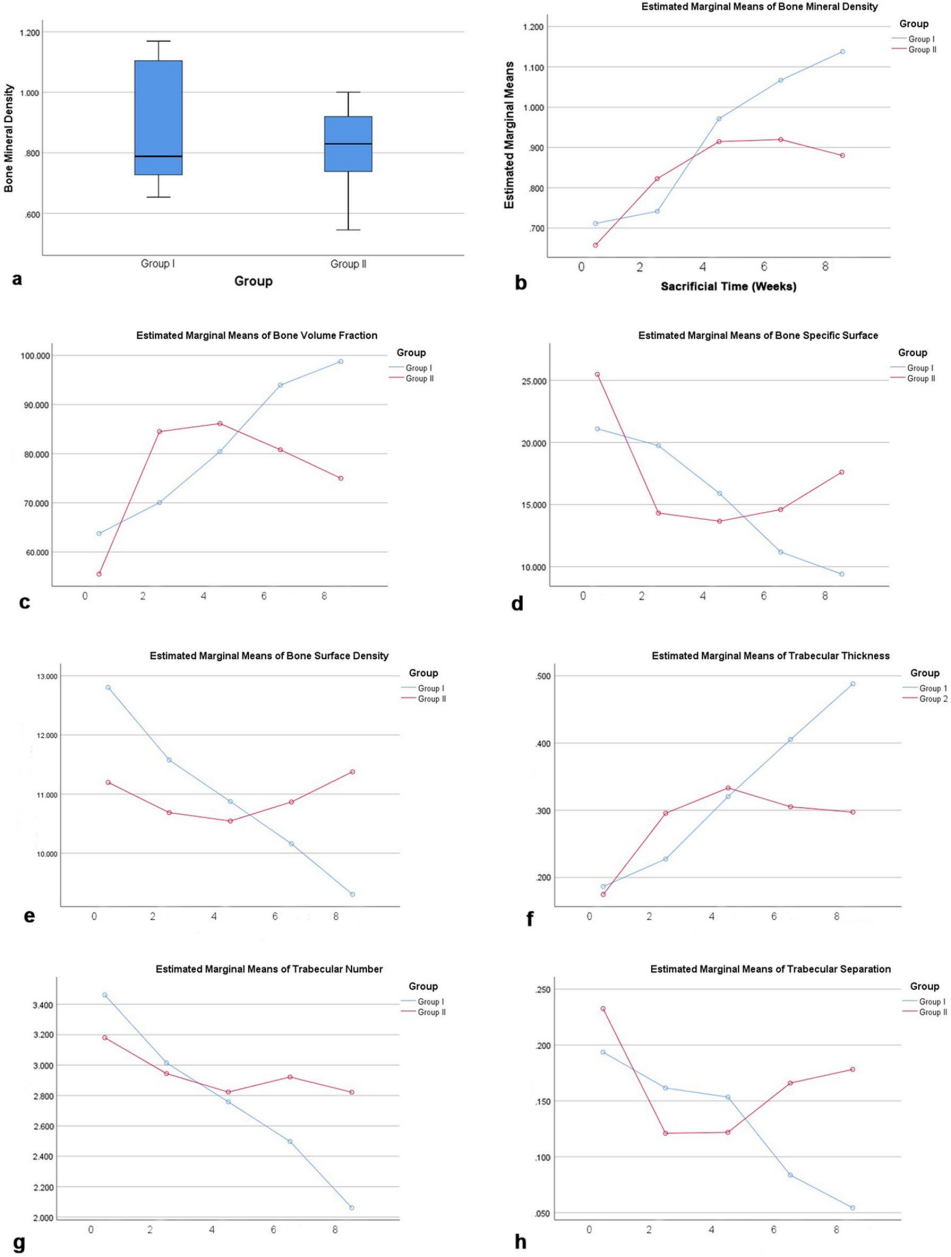
Changes in BMD and other bone morphometry parameter values over time in IP BRONJ model groups I and II. Group I shows higher BMD than group II (**a**). Group I shows a constant elevation of BMD compared with group II (**b**). Group I shows constant elevation in BV/TV (**c**) and Tb.Th. (**f**) and constant reduction in BS/BV (**d**), BS/TV (**e**), Tb.N. (**g**), and Tb.Sp. (**h**) compared with group II, in which the BP was stopped pre-extraction.

The statistical analysis of BMD and bone morphometry within group I showed significant differences in all parameters (*p* < 0.05), whereas group II showed a significant difference only in Tb.Th. (Table 5). The overall comparison showed higher BMD, BV/TV, BS/TV, and Tb.Th. values in group I than in group II (Table 5). Over time, group I showed constant changes in BMD and bone morphometry parameters compared to group II (Fig. 5). In group I, the BMD, BV/TV, and Tb.Th. values increased (Fig. 5b,c,f) and the BS/BV, BS/TV, Tb.N., and Tb.Sp. values decreased constantly. In group II, BS/BV, BS/TV, and Tb.Sp. values increased in the subgroups that were sacrificed at 6 and 8 weeks (Fig. 5d,e,h). The statistical analysis compared groups I and II over time until sacrifice at immediate, early (2 weeks and 4 weeks), and late (6 weeks and 8 weeks) post-extraction. Significant differences in BMD and bone morphometry were found between the groups that were sacrificed at a later period (*p* < 0.05). Group I showed significantly higher BMD, BV/TV, and Tb.Th. (1.102 ± 0.057 g/cm^3^, 96.359 ± 4.350%, and 0.447 ± 0.062 mm, respectively, *p* < 0.05) than group II. Furthermore, group II showed significantly higher BS/BV (16.101 ± 4.023 mm), BS/TV (11.121 ± 0.571 mm), Tb.N. (2.871 ± 0.263 mm), and Tb.Sp. (0.172 ± 0.029 mm) than group I (Table 6).

**Table 5:** Within group comparison and intergroup overall mean comparison of BMD and bone morphometry in the IP BRONJ model.

Group	BMD (g/cm^3^)	BV/TV (%)	BS/BV (1/mm)	BS/TV (1/mm)	Tb.Th. (mm)	Tb.N. (1/mm)	Tb.Sp. (mm)
***p*-value**							
I	0.001*	0.002*	0.007*	0.001*	0.000*	0.005*	0.001*
II	0.097	0.075	0.110	0.770	0.029**	0.772	0.096
**Mean ± SD**							
I	0.869 ± 0.192	77.257 ± 15.130	16.879 ± 5.338	11.299 ± 1.360	0.292 ± 0.117	2.896 ± 0.552	0.143 ± 0.056
II	0.809 ± 0.146	72.913 ± 16.435	18.523 ± 6.744	10.977 ± 0.693	0.263 ± 0.771	2.979 ± 0.347	0.175 ± 0.0598

The statistical analysis was performed using ANOVA for within group comparison. The data are depicted as *p-*values for within group comparison and mean ± standard deviation for intergroup overall comparison. *BMD* bone mineral density, *BV/TV* bone volume/total volume (bone volume fraction), *BS/BV* bone surface/segmented bone volume (specific bone surface), *BS/TV* bone surface/total volume (bone surface density), *Tb.Th.* trabecular thickness, *Tb.N.* trabecular number, *Tb.Sp.* trabecular separation. *Significant differences in BMD and bone morphometry parameters were found within group I (*p* < 0.05). **Tb.Th. differed significantly within group II (*p* < 0.05).

**Table 6:** Comparison of BMD and bone morphometry parameters between IP BRONJ model groups I and II based on time post-extraction.

Time Post-extraction	Group	BMD (g/cm^3^)	BV/TV (%)	BS/BV (1/mm)	BS/TV (1/mm)	Tb.Th. (mm)	Tb.N. (1/mm)	Tb.Sp. (mm)
Immediate (0 weeks)	I	0.712 ± 0.402	63.766 ± 1.178	21.095 ± 1.523	12.803 ± 0.898	0.187 ± 0.016	3.462 ± 0.331	0.194 ± 0.009
	II	0.658 ± 0.124	55.510 ± 12.549	25.481 ± 6.607	11.196 ± 0.980	0.175 ± 0.028	3.180 ± 0.475	0.233 ± 0.047
Early (2 and 4 weeks)	I	0.818 ± 0.163	73.517 ± 11.344	18.460 ± 4.417	11.342 ± 0.552	0.258 ± 0.06	2.929 ± 0.310	0.159 ± 0.269
	II	0.869 ± 0.081	85.321 ± 10.029	13.988 ± 2.670	10.615 ± 0.453	0.314 ± 0.034	2.883 ± 0.256	0.121 ± 0.043
Late (6 and 8 weeks)	I	1.102 ± 0.057*	96.359 ± 4.350*	10.293 ± 1.442	9.730 ± 0.626	0.447 ± 0.062*	2.279 ± 0.336	0.069 ± 0.0340
	II	0.900 ± 0.101	77.908 ± 9.486	16.101 ± 4.023**	11.121 ± 0.571**	0.301 ± 0.633	2.871 ± 0.263**	0.172 ± 0.029**

The statistical analysis was performed using student t testing. The data are depicted as the mean ± standard deviation. *BMD* bone mineral density, *BV/TV* bone volume/total volume (bone volume fraction), *BS/BV* bone surface/segmented bone volume (specific bone surface), *BS/TV* bone surface/total volume (bone surface density), *Tb.Th.* trabecular thickness, *Tb.N.* trabecular number, *Tb.Sp.* trabecular separation. *Group I was significantly higher than group II in BMD, BV/TV, and Tb.Th. (*p* < 0.05). **Group II was significantly higher than group I in BS/BV, BS/TV, Tb.N., and Tb.Sp. (*p* < 0.05).

### Histological and immunohistochemical results of medication discontinuation

#### Medication discontinuation in the alendronate oral medication model

**Figure 6:**
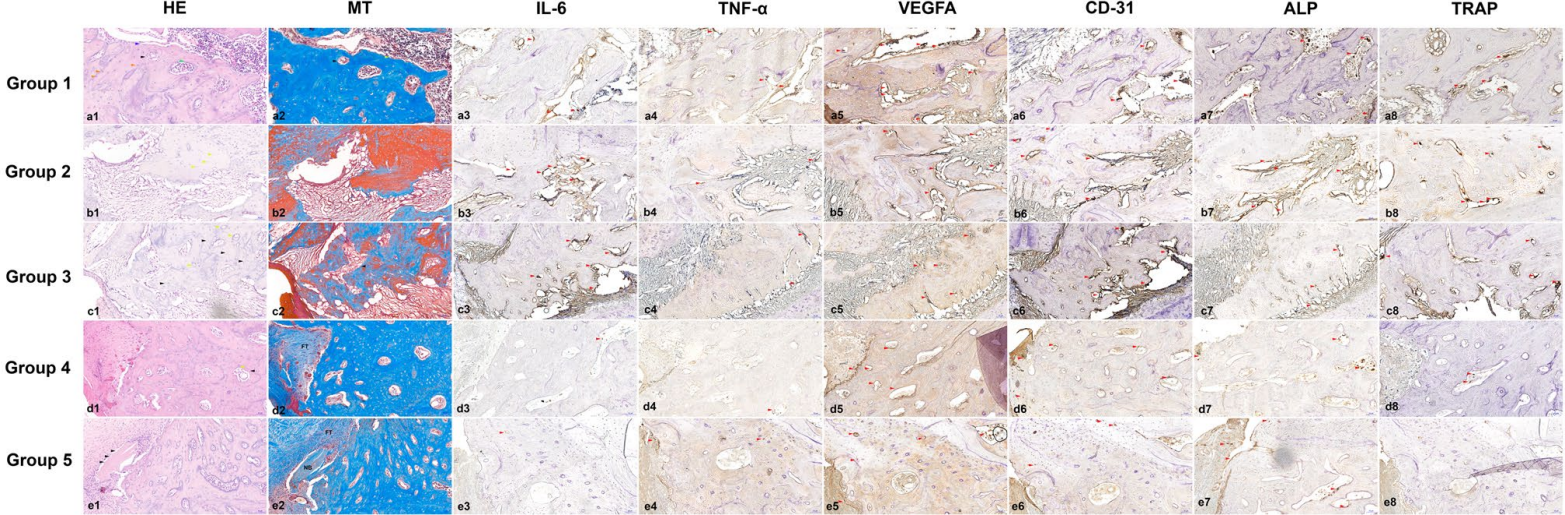
Representative histological and immunohistochemistry images from the PO BRONJ model at ×20 magnification, 50 μm. The H&E-stained slide from group 1 shows healthy osteocytes (orange arrowheads), osteoblast lining (blue arrowhead), Haversian canal (black arrowhead), and the incremental line of the bone (green arrowhead) (**a1**). In the MT staining, newly formed bone composed of abundant healthy osteocytes with a Haversian canal (black arrowhead) and osteoblast lining (yellow arrowhead) are visible (**a2**). Several pyknotic osteocytes (yellow arrowheads) appear in the H&E-stained slide from group 2 (**b1**). Connective tissues are visible (**b2**). Several empty lacunae (black arrowheads) and empty Haversian canals (yellow arrowheads) are visible in the H&E-stained slide from group 3 (**c1**). Thinning of the osteoblast lining (black arrowhead) is visible in the MT-stained slide from group 3 (**c2**). In group 4, the bone is filled with a mix of healthy osteocytes, pyknotic osteocytes, and empty lacunae. An area of bone resorption (black arrowhead) indicating osteon reconstruction is characterized by the osteoblast lining at the periphery (yellow arrowhead) and loose connective tissue in the center (**d1**). Fibrous tissue in the extraction socket is visible (**d2**). In group 5, bone composed of a mix of healthy osteocytes, pyknotic osteocytes, and empty lacunae can be seen. Below the fibrous tissue, an area of nonvital bone composed of empty lacunae (black arrowheads) is visible (**e1**). Bone composed of a mix of healthy osteocytes, pyknotic osteocytes, and empty lacunae can be seen. Below the fibrous tissue, an area of nonvital bone composed of empty lacunae is visible (**e2**). In immunohistochemistry images, red arrows indicate antibody expression. In terms of inflammation-related markers, the acute phase was shown with 3 weeks of administration, and the long-term effects of alendronate, low expression of IL-6, were shown upon 8 weeks of administration. VEGFA expression was suppressed by alendronate in the experimental groups. The attached-osteoclast ratio expressed by TRAP decreased over time. CD31 and ALP were moderately expressed in all groups. *FT* fibrous tissue, *NB* nonvital bone.

In group 1, the newly formed bone was composed of healthy osteocytes. Near the newly formed bone, a Haversian canal and osteoblastic lining surrounded by inflammatory infiltrates were observed (Fig. 6a1–2). In group 2, some pyknotic osteocytes and empty lacunae began to appear in the bone. In the MT staining results, connective tissue was observed in group 2 (Fig. 6b1–2).

The histological slides from group 3 showed several empty lacunae and an empty Haversian canal, with thinning of the osteoblastic lining (Fig. 6c1–2). Meanwhile, the histological features in group 4 revealed areas of bone resorption with osteoblast layers and loose connective tissue in the center. This area is involved in osteon reconstruction. Fibrous tissue formation was observed in the extraction socket (Fig. 6d1–2).

In group 5, the bone area was dominated by healthy osteocytes and a Haversian canal. However, on top of the bone area is a small area of nonvital bone covered by fibrotic tissue (Fig. 6e1–2). In quantitative analysis, the NBI percentage was highest in group 3 (35.417 ± 2.946%) and lowest in group 1 (27.180 ± 2.860%). However, that difference was not statistically significant (Table 7).

**Table 7:** Comparison of necrotic bone indicator percentage among the PO BRONJ model groups.

Group	Necrotic Bone Indicator (%)
Group 1	27.180 ± 2.860
Group 2	33.578 ± 0.804
Group 3	35.417 ± 2.946
Group 4	31.957 ± 2.644
Group 5	27.418 ± 5.363
*p*-value	0.064

The statistical analysis was performed using ANOVA. The data are depicted as the mean ± standard deviation.

The IL-6 expression was moderate in groups 1 (IRS 4), 2 (6), and 3 (6). In groups 4 and 5, the expression was mild (IRS 2) (Fig. 6a3–e3). TNF-α was moderately stained in group 1 (IRS 4) and mildly stained in groups 2 (IRS 3), 3 (3), 4 (2), and 5 (3). VEGFA expression was strongly positive in group 1 (IRS 9) and moderate in the other groups (IRS 6). CD31 was moderately expressed in all groups (IRS 4 for groups 1 and 5; IRS 6 for groups 2, 3, and 4). In TRAP staining, group 1 showed the highest number of osteoclasts (10 attached and 6 detached osteoclasts); group 2 showed 5 attached and 2 detached osteoclasts; group 3 showed 7 attached and 7 detached osteoclasts; group 4 showed 5 detached osteoclasts; and group 5 showed no osteoclasts. ALP was moderately stained in all groups (IRS 4 for groups 3 and 4; 6 for all other groups) (Fig. 6).

#### Medication discontinuation in the pamidronate intraperitoneal injection model

**Figure 7:**
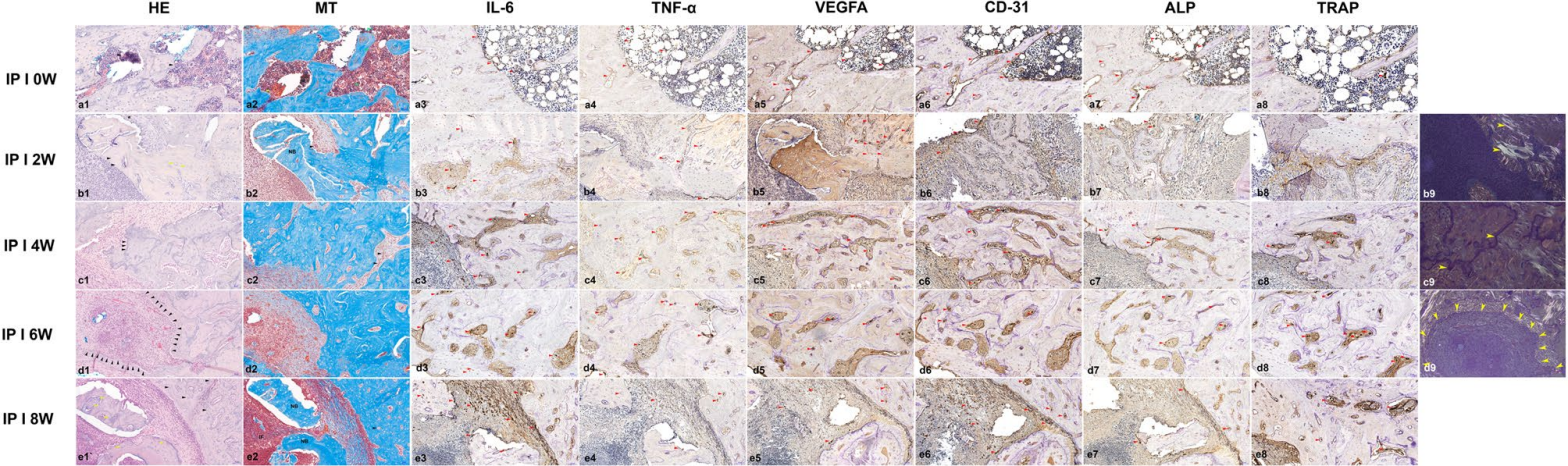
Representative histological and immunohistochemistry images from IP BRONJ model group I at ×20 magnification, 50 μm. In the H&E-stained slide of week 0 post-extraction, healthy osteocytes and abundant inflammatory infiltrate (black arrowheads) are visible (**a1**). In the MT staining week 0 post-extraction, abundant inflammatory infiltrate, a blood clot (yellow arrowhead), and detached osteoclasts (green arrowheads) are visible (**a2**). In the H&E-stained slide of 2 weeks post-extraction, empty lacunae (black arrowheads) and empty Haversian canals (yellow arrowheads) can be seen in the nonvital bone, ×20 magnification, 50 μm (**b1**). Nonvital bone is visible below the inflammatory infiltrate area. A detached osteoclast is also visible in the connective tissue (black arrowhead) (**b2**). At 4 weeks post-extraction, in the H&E-stained slide, an area of nonvital bone composed of empty lacunae (black arrowheads) is visible (**c1**). Newly formed collagen stained red (black arrowheads) is visible (**c2**). In representative slide images taken 6 weeks post-extraction, an area of abscess is visible (**d1**,**d2**). In representative slide images taken 8 weeks post-extraction, a nonvital bone area composed of mixed empty lacunae (yellow arrowheads) and pyknotic osteocytes (black arrowheads) is visible (**e1**). In the MT staining, an empty blood vessel is visible (**e2**). In immunohistochemistry images, red arrows indicate antibody expression. The expression of inflammatory-related antibodies was elevated from moderate to strongly positive over time. VEGFA expression decreased at 6 and 8 weeks post-extraction. ALP expression decreased over time, and more detached osteoclasts were observed over time in the TRAP staining. Under the polarized light microscope, samples taken 2 weeks post-extraction showing collagen fibers with bright birefringence (yellow arrowheads) (**b9**). Representative samples taken 4 weeks post-extraction showing the thick reversal line surrounding the area of nonvital bone with no birefringence (yellow arrowheads) (**c9**). Representative samples taken 6 weeks post-extraction showing an area of abscess with no birefringence (**d9**). *NB* nonvital bone.

In group I, several signs of BRONJ were observed. At week 0, the bone was dominated by healthy osteocytes and a healthy osteoblast lining. Inflammatory infiltration and blood clotting were observed, indicating wound healing. However, detached osteoclasts and a thick reversal line began to appear (Fig. 7a1–2). At 2 weeks, nonvital bone composed of empty lacunae with a thick reversal line and detached osteoclasts in the connective tissue was observed (Fig. 7b1–2). Under a polarized light microscope, collagen fibers with bright birefringence were observed (Fig. 7b9). At 4 weeks, new collagen fibers had formed. In some areas, nonvital bone composed of empty lacunae was also observed (Fig. 7c1–2). Under a polarized light microscope, a thick reversal line surrounded the area of nonvital bone with no birefringence (Fig. 7c9). At week 6, an area of abscess was observed at 2 × magnification, 50 μm (Fig. 7d1–2). The area of abscess showed no birefringence under a polarized light microscope (Fig. 7d9). At 8 weeks, empty blood vessels and a nonvital bone area composed of pyknotic osteocytes and empty lacunae were observed (Fig. 7e1–2).

**Figure 8:**
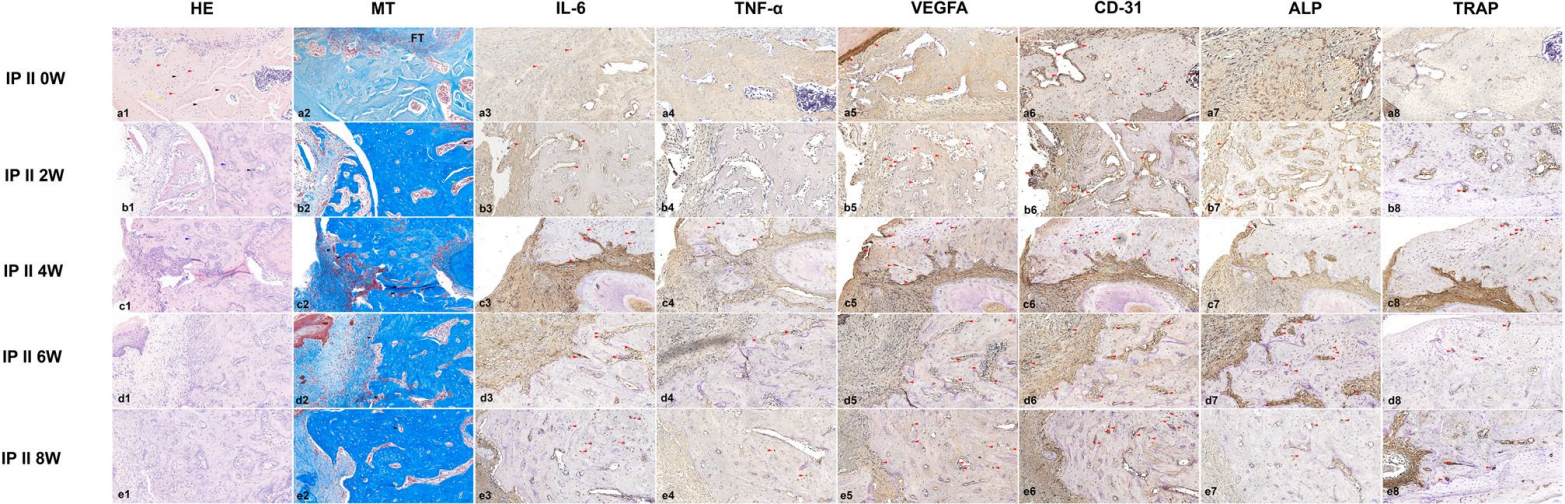
Representative histological and immunohistochemistry images from IP BRONJ model group II at ×20 magnification, 50 μm. In representative slide images taken at 0 weeks post-extraction, the bone was dominated by healthy osteocytes (red arrowheads), with some pyknotic osteocytes (yellow arrowheads) and empty lacunae (black arrowheads) also visible (**a1**). The MT-stained slide shows the bone adjacent to the fibrous tissue (**a2**). In the H&E-stained slide, 2 weeks post-extraction, the bone area was dominated by healthy osteocytes, with some pyknotic osteocytes and empty lacunae. In addition, a thinner reversal line was observed compared with group I (blue arrowheads), intact osteoclasts in the sealing zone (black arrowheads) and small sequestrum (red arrowheads) are also visible (**b1**). In the MT-stained slide, an osteoblast (yellow arrowhead) and vital blood vessel (black arrowhead) are visible (**b2**). In the H&E-stained slide at 4 weeks post-extraction, the bone area was dominated by healthy osteocytes with some empty lacunae. A reversal line (blue arrowhead) was also observed, ×20 magnification, 50 μm (**c1**). Intact osteoclasts in the sealing zone (black arrowheads) and a detached osteoclast with a wide Howship’s lacuna from the bone (green arrowhead) are visible (**c2**). At 6 weeks post-extraction, the bone area was dominated by healthy osteocytes, with some pyknotic osteocytes and empty lacunae, ×20 magnification, 50 μm (**d1**). Intact osteoclasts in the sealing zone (black arrowheads) and a detached osteoclast with a wide Howship’s lacuna from the bone (green arrowhead) are visible (**d2**). At 8 weeks post-extraction, the bone area was dominated by healthy osteocytes, with some pyknotic osteocytes and empty lacunae, ×20 magnification, 50 μm (**e1**). In the MT-stained slide, several blood vessels with blood clots inside (black arrowhead) (**e2**). In immunohistochemistry images, red arrows indicate antibody expression. The expression of inflammatory-related markers was mild to moderate over time. The angiogenesis antibody expression was moderate to strongly positive at all time points, and the recovery of angiogenesis was observed 8 weeks post-extraction. More attached osteoclasts were found over time, and ALP was moderately expressed at most times but strongly positive at 6 weeks post-extraction.

We also analyzed the histologic characteristics of group II. At week 0, the bone area was dominated by healthy osteocytes (Fig. 8a1–2). At week 2, the bone area was dominated by healthy osteocytes mixed with pyknotic osteocytes and empty lacunae. In addition, healthier osteoclasts and osteoblasts and a thinner reversal line were present compared with group I (Fig. 8b1–2). At week 4, the bone area was dominated by healthy osteocytes, although some empty lacunae were present, and intact osteoclasts remained in the sealing zone in the MT staining (Fig. 8c1–2). At week 6, intact osteoclasts persisted in the sealing zone. The bone area was dominated by healthy osteocytes, although some pyknotic osteocytes were also observed (Fig. 8d1–2). At 8 weeks, the bone area was dominated by healthy osteocytes, and blood vessels were found to contain blood clots (Fig. 8e1–2).

In quantitative analysis, the NBI percentage was highest in the 2-week experimental group both in groups I and II (74.815%, 42.332 ± 5.532%, respectively) (Table 8). The comparison of NBI percentage among the subgroups within group I showed significant differences (*p* < 0.05), with an increasing trend over time starting at 4 weeks post-extraction (Table 8). The comparison of NBI percentage among the subgroups within group II showed no significant difference, with a decreasing trend over time starting at 2 weeks post-extraction (Table 8). The comparison of the NBI percentage between group I and II experimental subgroups showed significant differences, with group I showing a higher NBI percentage (51.926 ± 12.749%) than group II (34.082 ± 7.972%) (*p* < 0.05) (Table 8).

**Table 8:** Comparison of necrotic bone indicator percentage (NBI) in IP BRONJ model group I and II over time.

Group	Time post-extraction	NBI (%)	Total NBI (%)	*p*-value
I	0 weeks	19.116 ± 0.527		
	2 weeks	74.815		
	4 weeks	36.986	51.926 ± 12.749*	0.003**
	6 weeks	48.5671 ± 3.015		
	8 weeks	54.054		
II	0 weeks	11.039		
	2 weeks	42.332 ± 5.532		
	4 weeks	33.927 ± 8.626	34.082 ± 7.972	0.154
	6 weeks	32.039 ± 2.039		
	8 weeks	28.032 ± 2.106		

The statistical analysis was performed using ANOVA. The data are depicted as the mean ± standard deviation. *The NBI percentage in group I was significantly higher than that in group II (*p* < 0.05). **Significant differences in necrotic bone indicator percentage were found within group I (*p* < 0.05).

In group I, the expression of IL-6 was moderate at week 0 (IRS 4) and week 2 (IRS 6) and strongly positive at 4, 6, and 8 weeks (IRS 9). All subgroups showed moderate TNF-α (IRS 6 at 0, 2, and 8 weeks; IRS 8 at 4 and 6 weeks). The VEGFA level was moderate at 0, 6, and 8 weeks (IRS 6) but strongly positive at 2 and 4 weeks (IRS 9). CD31 was moderate at 0, 4, and 6 weeks (IRS 6); moderate at 2 weeks (IRS 4); and strongly positive at 8 weeks (IRS 9). In TRAP staining, 1 detached osteoclast was found at week 0; none were found at 2 weeks; 13 detached and 8 attached osteoclasts were found at 4 weeks; the highest numbers of osteoclasts, 22 detached and 10 attached, were found at 6 weeks; and 13 attached and 11 detached osteoclasts were found at 8 weeks. The highest level of ALP expression (IRS 9) was found at week 0; the rest of the groups showed a moderate expression (IRS 4) at 2, 6, and 8 weeks and of 6 at 4 weeks (Fig. 7).

In group II, mild expression of IL-6 was shown at week 0 (IRS 3), whereas the other groups showed a moderate expression (IRS 6). At 2 weeks, TNF-α expression was negative (IRS 1), whereas expression was mild at weeks 0 (IRS 2) and 6 (IRS 3) and moderate at 4 and 8 weeks (4). VEGFA expression was strongly positive at 4 and 8 weeks (IRS 9) and moderate at weeks 0 (IRS 6) and 2 and 6 weeks (IRS 8). CD31 expression was strongly positive in all subgroups (IRS 9). In TRAP staining, 5 attached osteoclasts were found at week 8; 3 attached osteoclasts were found at week 6; 9 attached and 3 detached osteoclasts were found at 4 weeks; 1 detached osteoclast was found at 2 weeks; and no osteoclasts were found at week 0. ALP expression was strongly positive at 6 weeks (IRS 9) and moderate in the other subgroups: IRS 6 at weeks 2 and 4 and IRS 4 at weeks 0 and 8 (Fig. 8).

## Discussion

The results of the current study rejected the null hypothesis by showing that the discontinuation of medication significantly suppressed the adverse effects of BP on alveolar bone including the remodeling process based on the micro-CT, histological, and immunohistochemical analysis. In the previous clinical study, none of the 101 subjects who discontinued BP before tooth extraction developed BRONJ, although delayed wound healing was observed in two patients^33^. The discontinuation of BPs is reported to recover bone remodeling, osteoclasts and precursors, and bone marrow function, indicated by increased upregulation of the serum C-terminal telopeptide biomarker^34^. To the best of our knowledge, the current study analyzes for the first time the significance of the discontinuation of medication in both the oral and IP administration of BPs in BRONJ models in vivo.

The medication selection and dose used in the establishment of the two BRONJ models in this study are based on the guidelines for developing osteonecrosis in the rat jaw^35,36^. The discontinuation of medication resulted in comparable outcomes to the group with continuous medication in terms of BRONJ severity. The significance of this study is that it demonstrates the effectiveness of discontinuing medication in a BRONJ model by significant bone remodeling recovery effects based on micro-CT, histology, and IHC analyses.

We demonstrate our methodology according to the standard guideline for bone microarchitecture analysis in rats using micro-CT^37^. Because BP deposition is higher in active resorption sites, a larger proportion of each BP dose is taken up by trabecular bone than by cortical bone^35,38^. The important parameters in evaluating the effects of BPs on alveolar bone are BMD, BV/TV, BS/BV, Tb.Th., Tb.N., and Tb.Sp. In the PO BRONJ model, no significant differences in BMD or bone morphometry parameters were found between the control and experimental groups, although group 3 showed the highest BMD, BS/TV, and Tb.N. values. In the IP BRONJ model group I, the BMD, BV/TV, and Tb.Th. values significantly increased and the BS/BV, BS/TV, Tb.N., and Tb.Sp. values significantly decreased over time (*p* < 0.05). Combining BPs with a steroid, such as dexamethasone, could intensify the repressive effect that BPs have on bone resorption, which leads to increases in BMD, Tb.Th., and BV/TV^39^. The BV/TV parameter shows extended ossification, also called over-ossification^40,41^. The over-ossification of bone decreases the soft tissue volume and thereby obstructs the blood supply by occupying space that is essential for physiologic vascularization. It also disorganizes the trabecular microarchitecture. The increase in bone mass and disorganized trabecular bone formation poses a high risk of malnourishment and microcracks and consequently easier access for pathogens^41^. In contrast to group I, group II showed an increasing trend in BS/BV, BS/TV, and Tb.Sp. and a decreasing trend in BMD and BV/TV, in the subgroups sacrificed at 6 and 8 weeks. As the values of BS/BV and BS/TV increase, the remodeling rate becomes higher, and the mineral density becomes lower due to the increased formation and presence of osteoid^42^. These parameters are associated with the propensity of tissue to renew and adapt its microstructure by initiating bone modeling and remodeling events. They also define the density of the surfaces upon which osteoclasts and osteoblasts can perform their bone-resorbing and bone-forming actions and further describe the local surface area through which biochemical signals transduced by osteocytes from local mechanical signals can be transmitted to cells in the marrow and vascular spaces^43^.

Our micro-CT results were supported by the histological analysis. The group without discontinuation showed signs of early BP toxicity. In a previous study, BP-involved bones are described as immature bony matrices outlined by thick reversal lines that are crucial to the rapid osteonecrosis of BRONJ, and under a polarized light microscope, these areas show no birefringence^44^. The group with BP discontinuation continued to show numerous healthy osteocytes, intact osteoclasts in the sealing zone, and vital blood vessels, which are essential for the remodeling process. The IHC results corresponded with the micro-CT results and showed a higher IRS in bone remodeling-related proteins, TRAP, and ALP in groups that discontinued medication compared to those with continuous medication.

TNF-α, IL-6, and IL-1 are three major cytokines that play important roles in the inflammatory response^45^. TNF-α activates antimicrobial pathways critical for host defense and IL-6 is important in protecting host cells from apoptosis^46^. In BRONJ, the nitrogen BPs inhibit the mevalonate pathway and prompt the production of TNF-α and other inflammatory markers by macrophages and monocytes. The TNF-α then heightens the generation of IL-6. The heightened level of inflammatory cytokines especially TNF-α, IL-β, and high oxidative stress from inducible nitric oxide synthase in BRONJ interrupts regular bone turnover by modulating osteocyte apoptosis^47,48^. In the acute phase, alendronate enhances IL-1β and IL-6 with only a slight effect on TNF-α and later inhibits osteocyte apoptosis and inflammation by inhibiting IL-6^49,50^. In our PO model, there was no difference in the TNF-α IRS among groups but IL-6 expression was elevated by 3 weeks of alendronate administration and reduced by 8 weeks, compared with the control. This indicates that the acute phase of alendronate action on bone in rats occurs in the first 3 weeks of administration. The administration of alendronate for 8 weeks showed the effects of long-term use in inhibiting IL-6 expression. In correlation with the presence of osteoclasts and empty lacunae, the rats in groups 2 and 3 had a higher NBI percentage than the other groups and attached osteoclasts could be observed. The NBI scores in groups 4 and 5 were lower than those in groups 2 and 3 and similar to that in group 1 but group 4 showed 5 detached osteoclasts, and group 5 showed no osteoclasts. In the IP model, IL-6 expression was elevated over time in both groups with higher expression and more detached osteoclasts in group I, indicating that pamidronate elevates IL-6 levels. The IRS of TNF-α and NBI was higher in group I than group II confirming that discontinuing BPs reduces the overexpression of TNF-α and can lead to cell injury and osteocyte apoptosis caused by pamidronate which is in accordance with our micro-CT results that showed an increasing trend in bone remodeling parameters. When the PO and IP model results are considered together, they confirm that the IP BPs had higher potency than the oral BPs.

Previous studies have found that pamidronate inhibited angiogenesis by reducing VEGFA and CD31 expression in murine macrophage cells, which led to the development of BRONJ^13,51,52^. Alendronate inhibits VEGFA expression through mevalonate pathway inhibition^53^. In our PO model, VEGFA expression was more strongly positive in the control than in other groups, indicating the suppression of angiogenesis by alendronate. Among the IP groups, group II had a higher IRS than group I for both VEGFA and CD31. In the 2-week subgroups of group I, CD31 was moderately expressed, whereas in those of group II, CD31 expression was strongly positive. VEGFA was more strongly positive in group II at 8 weeks than it was in group I. The lower expression of angiogenesis markers in group I was caused by over-ossification as shown in the micro-CT result. CD31 is widely accepted as a marker of vascularization, including microvessels, and is regarded as an endothelial progenitor. VEGFA is a growth factor imperative to angiogenesis, vasculogenesis, epithelization, and collagen deposition, especially in wound healing, and plays a role in bone remodeling by inducing osteoclast differentiation in monocytes^52,54^. Therefore, the expression of VEGFA is also implicated in the expression of TRAP which was in accordance with our results.

TRAP is a metalloenzyme used as an osteoclast marker that correlates positively with bone resorption, and TRAP-deficient mice have increased bone density^55^. In an earlier BRONJ model, TRAP expression decreased in BP-treated mice compared with saline-treated mice^56^. In this study, the PO group showed a decreasing trend in the attached-osteoclast ratio over time. More detached osteoclasts were found in IP group I at 4, 6, and 8 weeks compared with the PO model and IP group II. A previous study showed that pamidronate suppresses osteoclastogenesis by modifying genes vital to bone turnover^13^. Group II showed more attached osteoclasts in the 6- and 8-week subgroups, indicating the recovery of healthy osteoclasts over time.

ALP is an osteoblast differentiation marker expressed in the early stage of osteoblast differentiation and persists in early and mature osteoblasts, which can be reduced by BP intervention^57^. In the PO and IP models, ALP expression was moderate. However, ALP expression in IP group I decreased over time compared with group II. In group II the expression of ALP fluctuated over time. The effects of pamidronate in altering osteoblast activity seemed to be reduced when BP administration was discontinued before tooth extraction and persisted when BP administration was continued.

The results were in accordance with our hypothesis that discontinuing medication reduces BRONJ severity following tooth extraction in rats, and the null hypothesis is rejected. With discontinuation of medication for at least one week in rats, the effects of BPs on alveolar bone are suppressed, and bone turnover and osteoclast functions are restored.

Considering that most tooth extractions that are performed in humans involve infected teeth, the major limitation of this study is the absence of infection for development of BRONJ. An infected tooth in rats may affect the severity of BRONJ, and the efficacy of BP discontinuation based on BRONJ severity can be scrutinized further. Another limitation is the absence of a continuously medicated group in the PO model. With the presence of this group in the PO model, a direct comparison of relevant variables could be made with the BP discontinued group.

In the future, the current model can be enhanced by addition of various periods of medication discontinuation, for studying the different outcomes in micro-CT and histologic analyses in relation to duration of medication discontinuation. In this way, the duration of medication discontinuation can be determined and standardized. Within the limitations of this study, we demonstrate the significance of discontinuing medication on BRONJ in a rat model.

## Data Availability

The datasets generated during and/or analyzed by the authors during this study are available from the corresponding author on reasonable request.
